# Modeling the Production Process of Lignin Nanoparticles Through Anti-Solvent Precipitation for Properties Prediction

**DOI:** 10.3390/nano14221786

**Published:** 2024-11-06

**Authors:** Victor Girard, Laurent Marchal-Heussler, Hubert Chapuis, Nicolas Brosse, Nadia Canilho, Isabelle Ziegler-Devin

**Affiliations:** 1 LERMAB, Faculty of Science and Technology, University of Lorraine, INRAe, F-54000 Nancy, France; hubert.chapuis@univ-lorraine.fr (H.C.); nicolas.brosse@univ-lorraine.fr (N.B.); 2 ENSIC, University of Lorraine, F-54000 Nancy, France; laurent.marchal-heussler@univ-lorraine.fr; 3 L2CM, Faculty of Science and Technology, University of Lorraine, CNRS, F-54000 Nancy, France; nadia.canilho@univ-lorraine.fr

**Keywords:** lignin, nanoparticles, organosolv process, prediction model, anti-solvent precipitation

## Abstract

Global warming has recently intensified research interest in renewable polymer chemistry, with significant attention directed towards lignin nanoparticle (LNP) synthesis. Despite progress, LNP industrial application faces challenges: (1) reliance on kraft lignin from declining raw biomass processes, (2) sulfur-rich and condensed lignin use, (3) complex lignin macroparticles to LNP conversion, using harmful and toxic solvents, and, above all, (4) lack of control over the LNP production process (i.e., anti-solvent precipitation parameters), resulting in excessive variability in properties. In this work, eco-friendly LNPs with tailored properties were produced from a semi-industrial organosolv process by studying anti-solvent precipitation variables. Using first a parametric and then a Fractional Factorial Design, predictions of LNP sizes and size distribution, as well as zeta-potential, were derived from a model over beech by-products organosolv lignin, depending on initial lignin concentration (x_1_, g/L), solvent flow rate (x_2_, mL/min), antisolvent composition (x_3_, H2O/EtOH *v*/*v*), antisolvent ratio (x_4_, solvent/antisolvent *v*/*v*), and antisolvent stirring speed (x_5_, rpm). This novel chemical engineering approach holds promise for overcoming the challenges inherent in industrial lignin nanoparticle production, thereby accelerating the valorization of lignin biopolymers for high value-added applications such as cosmetics (sunscreen or emulsion) and medicine (encapsulation, nanocarriers), a process currently constrained by significant limitations.

## 1. Introduction

According to a recent study from 2023, the chemical industry contributes approximately 5% to global CO_2_ emissions [1], securing its position as the third-largest emitter due to reliance on fossil fuels [2]. To address this issue, reinvention through innovations in raw materials and reaction engineering is needed to generate environmentally friendly products based on green chemistry principles.

One such approach is the holistic biorefineries model, where the entire lignocellulosic biomass (LCB) from waste is valorized into various high-value bioproducts using interesting eco-friendly processes. Currently, almost 70% of the LCB, consisting of cellulose and hemicelluloses polysaccharides [3], is efficiently valued into chemicals [4], biofuels [5], pulp [6], and fibers and nanofibers [7], while the remaining 20–30% is composed of lignin biopolymers [8], mainly serving as an energy source [9]. Indeed, due to its important binding with polysaccharides and complex chemical structure, lignin has often been seen as a critical barrier in biorefinery models and, therefore, is categorized as waste [4].

Lignin, the predominant aromatic biopolymer in nature [10], involves an amorphous and irregular shape mainly composed of three monomeric units, named p-hydroxyphenyl (H), guaiacyl (G), and syringyl (S), interconnected among others [11]. Considering the annual global production of biomass waste estimated at 140 Gt [12], lignin is an aromatic, renewable, biodegradable, and abundant material [13], making it a central resource in mitigating carbon emissions associated with traditional chemical processes.

Given the intricate complexity and heterogeneous nature of lignin’s structure, pretreatment technologies play a pivotal role in facilitating its applications, as is the case with the organosolv pulping method [14]. In a pressurized heated and agitated reactor, organosolv pretreatment involves LCB breakdown and lignin solubilization in organic solvents such as EtOH, providing environmental benefits by using non-toxic chemicals, minimizing emissions, reducing equipment corrosion, and ensuring low energy consumption through the recovery of high-volatility alcohol via simple distillation [15]. Unlike the kraft and sulfite processes of the conventional paper industry, which increase lignin dispersity [10], organosolv technology enables the extraction of sulfur-free and pure lignin with a poor condensed structure that closely matches the native one, opening up possibilities for improved valuations [16,17]. Furthermore, organosolv also improves cellulose isolation due to organic solvents, positioning this technology as a promising asset for the biorefinery model [18].

Positioned as the potential driving force behind the next industrial revolution, bio-based nanoscale technology may be an alternative approach to overcome lignin heterogeneity while offering a wide array of benefits tailored to meet evolving consumer needs across various sectors including energy, transportation, agriculture, food, materials, electronics, and medicine [19]. Recently, manipulating lignin at the nanoscale level has been considered to be a significant process for a sustainable biorefinery model [20]. Interestingly, reduced lignin size enhances both morphological and chemical uniformity, yielding advancements in its structural integrity and compositional consistency [21]. Due to their heightened surface area to volume ratio, LNPs exhibit distinctive adjustable and multifunctional properties, including improved antibacterial, anti-oxidant, and UV protection properties such as greater homogeneity [22,23]. The growing interest around LNPs has encouraged significant advancements across fields including energy, environment, materials, medicine, cosmetics, and food [24,25], owing to their advantages of biocompatibility, non-toxicity, medium-term biodegradability, environmental resistance, and improved properties [26,27]. The demonstrated low-cytotoxicity of LNPs has been crucial for generating industrial concern and ensuring the viability of lignin-based biorefinery models by opening up new opportunities in markets involving human contact [28].

LNPs can be synthesized using diverse methodologies, including assisted or self-assembly formation, with supercritical CO2 treatment [29], mechanical or ultrasonication homogenization [30,31,32], aerosol processing [33], electrospinning [34], ice segregation [35], cross-linking/polymerization [36], solvent exchange, and pH shifting [37,38,39,40]. The most common and promising technique for leveraging green chemistry approaches to industrial applications is based on the self-assembly of LNPs through the antisolvent precipitation [26]. This method also refers to the nanoprecipitation and ouzo effect [41] and relies on lignin dissolution into water-miscible solvents (e. g., ethanol, acetone, THF, and DMSO, among others) shifted with an excess amount of an antisolvent (e.g., water) [42]. LNP formation is facilitated through orderly rearrangement and aggregation of lignin macroparticles (LMPs), harnessing lignin’s amphiphilic nature and both hydrophilic and hydrophobic interactions with solvents driven, respectively, by aromatic structures and internal H-bonding [43].

Despite the numerous advantages offered by LNPs, their production and valorization remain significant industrial challenges due to the complexity of controlling multiple parameters in a large-scale process [17]. Indeed, in a previous study [44], Girard et al. reinforced earlier findings [16,43,45,46] that highlighted the significance of the inherent structures of lignin oligomers, impacted by both biomass nature (hardwood, softwood, and herbaceous material) and chemical extraction methodology, as a key factor affecting the final LNP’s properties (size, morphology, stability), and, therefore, the associated industrial valuations. It was demonstrated that the lignin self-assembly process was partly influenced by lignin molecular weight and its amphiphilic nature, intricately related to the content of phenolic and aliphatic units, the building unit type (H,G,S), and non-covalent interactions such as π–π interactions [47]. To this end, hardwood materials were identified as optimal feedstocks for LNP production, leading to enhanced nucleation characterized by formation of smaller and spherical particles compared to softwoods and herbaceous species [44]. Similarly, within the context of a specific biomass specie, the organosolv extraction method produced improved lignin structure without sulfur [44], leading to enhanced aggregation and greater LNP properties compared to other extraction methods such as the kraft process [48].

Then, other studies also demonstrated that the self-assembly process is also partly driven by the precipitation method itself, which depends on parameters such as antisolvent flow rate [37,39,49,50,51], initial lignin concentration [43,52,53], solvent temperature [52,54], solvent type [40,49,52,55], solvent ratio [43,49,52,56], stirring speed [51,52], and pH value [37,40,51]. However, a significant issue arises from the fact that the majority of previous studies have analyzed factors in isolation regarding the kraft lignin, which fails to adequately address process variability or serve as a basis for potential industrialization.

Hence, for the first time, in this work, our efforts are built upon previous findings to devise a controlled, environmentally friendly method using only water and ethanol for producing tailored and predictable LNPs from organosolv isolation. This approach may easily be transposed on an industrial scale to accelerate the development of value-added applications. To achieve this objective, LMPs were first extracted from beech by-products using s 10 L semi-industrial organosolv reactor, generating enough material for parametric studies. Then, a parametric study on LNP production from beech lignin using only water and ethanol was performed to define the relevant range of values in which each parameter can vary. This was followed by a fractional factorial design plan, including the following parameters studied in the parametric study: (1) initial lignin concentration (1–50 g/L), (2) solvent flow rate (0.5–500 mL/min), (3) antisolvent composition (100/0–50/50 H_2_O/EtOH *v*/*v*), (4) antisolvent volume (1/2–1/20 solvent/antisolvent *v*/*v*), and (5) antisolvent stirring speed (150–1200 rpm). The extracted LMPs underwent detailed characterization utilizing nuclear magnetic resonance spectroscopy (NMR) and size exclusion chromatography (SEC). The size distribution of LNPs was analyzed using dynamic light scattering (DLS) and transmission electron microscopy (TEM). This approach enabled us to both rank the impacts of the LNP manufacturing parameters and process parameters, as well as to predict LNP sizes, thanks to a model which relies on manufacturing parameters. These results can be used to design an environmentally friendly manufacturing process of lignin and LNPs at production scale.

## 2. Materials and Methods

### 2.1. Raw Materials and Reagents

Beech (Fagus sylvatica/Hardwood) by-products from a local forest (Grand Est Region, France) were used in this study. Water applied for lignin recovery and antisolvent precipitation was purified using Veolia Purelab^®^ Flex (Aubervillers, France) equipped with a 0.2 µm PES high-flux capsule filter (18.2 MΩ.cm at 23 °C). Ethanol (EtOH) used for organosolv was purchased from VWR^®^ chemicals (VWR International, Radnor, PA, USA). The chemical reagents utilized for characterizing lignin macroparticles were consistent with those detailed in a previous study [44].

### 2.2. Lignin Isolation with Organosolv Process

Lignin isolation from ø8 mm beech particles was achieved through organosolv pretreatment. Before extraction, the chemical composition of feedstock was characterized based on previous research conducted by Girard et al. [44] and is given in Table 1. Organosolv pretreatments were carried out using a tailor-made 10 L reactor Grayel et Fils^®^ (Lyon, France). The lignin isolation procedure was the same as previously detailed in Girard et al. [44]. In brief, 600 g of biomass (dry basis) was treated at a temperature of 200 °C in 40/60 aqueous/ethanol *v*/*v* mixture for 1 h with a 10/1 liquid/solid ratio. Following the pretreatment period, the reactor was rapidly cooled using a water circulation system, and the solid phase (cellulose rich pulp) was separated from the organosolv liquor (lignin and hemicellulose) through vacuum filtration. The cellulose-rich solid residue was washed twice with the mixture volume needed for the reaction during the filtration to ensure the thorough removal of residual impurities. Lignin recovery from an organosolv liquor using water precipitation, as well as associated isolation yield calculations, were achieved according to a previous work [44]. This entire procedure was performed 3 times for repeatability calculations.

### 2.3. Lignin Macroparticle (LMP) Characterization Properties

The isolated lignin from organosolv pretreatment was characterized using size exclusion chromatography (SEC) (Nara, Japan), phosphorus-31 and heteronuclear single quantum coherence (HSQC) nuclear magnetic resonance spectroscopy (NMR) (Bruker Scientific, Billerica, MA, USA). The results were compared with beech milled wood lignin (MWL) extracted according to the Qian et al. method [57]. The sulfur content of organosolv lignin was determined by elemental analysis using a Thermo Finnigan Flash EA^®^ 112 Series (Thermo Fisher Scientific, Waltham, MA, USA). A 1.5 mg sample was combusted at 1000 °C for 15 s under an oxidizing atmosphere with tungstic anhydride, and the resulting gas was reduced to N₂ using copper and then analyzed using gas chromatography. Detailed procedures for SEC and NMR are provided in the Supplementary Materials, following the methodology outlined in a previous work [44].

### 2.4. Lignin Nanoparticle (LNP) Synthesis and Experimental Design

This study first followed a meticulous cascade design, focusing on one parameter within its respective value range at a time. Aqueous ethanol antisolvent precipitation was used for LNP preparation. Based on previous work [44], lignin solutions with a range of concentrations (x_1_, 1–50 g/L) were prepared by dissolving LMPs in an 80/20 ethanol/water mixture, previously identified as the optimal ethanol concentration to solubilize lignin. Following an initial 1 h ultrasonic treatment to enhance solubilization, the lignin solutions were subsequently filtered through a 0.45 µm nylon filter to eliminate potential aggregates (less than 5 wt %), after which lignin solubilization yields were carefully determined.

**Figure 1:**
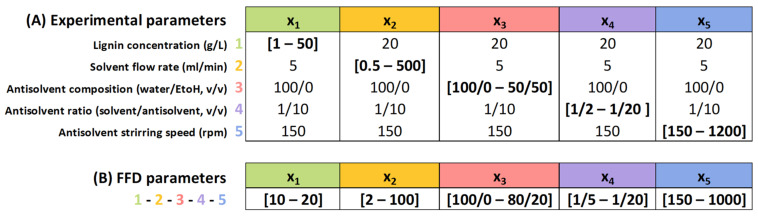
(**A**) Summary of the different experimental parameters used for LNP synthesis. xi represents the different variables (values in bold brackets) with x1 (initial lignin concentration, g/L), x2 (solvent flow rate, mL/min), x3 (antisolvent composition, water/EtOH, *v*/*v*), x4 (antisolvent ratio, solvent/antisolvent, *v*/*v*), and x5 (antisolvent stirring speed, rpm). (**B**) Summary of the different parameters used for the FFD model. xj represents the same variables as (**A**), but with different values (in bold brackets) to maintain the self-assembly mechanism during precipitation. The specific values for each variable for the screening design construction, along with the corresponding results, are presented in Figure S5.

In order to regulate the flow rate (x_2_) of the solvent flow across the study range of 0.5–500 mL/min, a KD Scientific^®^ Legato 200 syringe pump (KD Scientific, Holliston, MA, USA) was used (high rates over 200 mL/min were meticulously managed manually with volume/time conversion). Regarding antisolvent parameters, variations in antisolvent composition (x_3_, ranging from 100/0 to 50/50 H2O/EtOH *v*/*v*) were investigated, alongside adjustments in the dilution ratio (i.e., x_4_, antisolvent volume ratios from 1/2 to 1/20 solvent/antisolvent *v*/*v*). Additionally, the effect of the antisolvent stirring speed (x_5_, 150–1200 rpm) was also analyzed. Figure 1A summarizes the different parameters (x_i_) used (27 experiments).

Then, based on the results of the first method, a Fractional Factorial Design (FFD) including 2^5−1^ assays plus 1 central point (17 experiments) was conducted to evaluate the individual effect of independent same variables x_i_ (initial lignin concentration (x_1_, g/L), solvent flow rate (x_2_, ml/min), antisolvent composition (x_3_, H2O/EtOH *v*/*v*), antisolvent ratio (x_4_, solvent/antisolvent *v*/*v*), and antisolvent stirring speed (x_5_, rpm)) on a selected response (particle size, nm). In the FFD, careful consideration was given to selecting appropriate ranges for each variable to maintain the same precipitation and LNP assembly mechanism during precipitation. To achieve this, a constant temperature of 25 °C was maintained across all experiments. The specific values of the variables for each study are summarized in Figure 1B and Figure S5. For each experiment, triplicate trials were conducted to ensure repeatability. Significant variables were identified at a 10% significance level (*p*-value ≤ 0.10) using the experimental design, and the effects were generated using Minitab^®^ Statistical software (Version 21.1.0., State College, PA, USA).

### 2.5. LNP Characterizations

The particle’s size distribution, polydispersity index (PDI), and ζ-potential of the produced suspensions were analyzed using a Malvern^®^ Zetasizer ULTRA Dynamic Light Scattering (DLS) instrument (Grovewood, UK). The suspensions were analyzed immediately with post-precipitation with 1.5 mL at 25 °C in complete optical PS cells, without dilution. This approach offers an advantage over the existing literature, where a water dilution, evaporation, or solvent change step is often introduced before analysis, thereby introducing uncertainty regarding the influence of precipitation factors. Triplicate measurements were conducted in DLS mode at an angle of 174°. ζ-potential analyses were conducted under the same conditions, employing special folded capillary Zeta cells (DTS 1070) at 25 °C. Then, the suspensions were stored at 4 °C before further use.

A FEI Philips^®^ CM200 Transmission Electron Microscope (TEM, Amsterdam, The Netherlands) operating at an accelerating voltage of 160 kV was employed not only to correlate the DLS size measurements, but also to provide particle morphology information. Three suspensions from the cascade design study, representing both extremes and the central value, were investigated for each parameter to enhance the visualization of their impact on nanoparticle properties. For TEM analysis, each suspension was diluted to achieve a uniform final concentration of 10 mg/L. Finally, samples were directly prepared by applying a drop of LNPs suspension onto a TEM grid without contrasting agents, followed by drying for 30 min.

## 3. Results

### 3.1. LMP Isolation from Semi-Industrial Organosolv Reactor

Despite the well-known advantages of organosolv pretreatment over current industrial process such as kraft and soda (i.e., minimal use of toxic chemicals, organic solvent recovery, pure cellulose generation with efficient removal of pure (low ash and sugar content), and sulfur-free lignin from lignocellulosic biomass) [21], its application is limited by significant laboratory-scale constraints. Most studies use reactors that process small quantities of biomass (i.e., from 5 to 50 g in 0.1 to 1 L reactors) [14,16,58], yielding only a few grams of lignin. This limited output restricts further development and investigations of organosolv LNP processing, which requires large amounts of LMPs. In this study, a semi-industrial 10 L organosolv reactor is used to generate approximately 100 g of LMPs per batch, providing sufficient material for an in-depth investigation of nanoprocessing with this type of lignin.

As shown in Table 1, organosolv pretreatment without acid catalysis leads to an important chemical breakdown of the lignocellulosic biomass, significantly reducing both lignin and hemicellulose content through autohydrolysis and cleavage of α-/β-O-aryl ether and 4-O-methylglucuronic linkages. Lignin content in biomass residue after the reaction decreases from 23.7 to 13.0%, and hemicellulose reduces from 22.5 to 13.0%. As described by Brosse et al. [59], the organosolv process results in cellulose-rich pulp, as found in other investigations (51.5% compared to 47.8% initially) [57,60]. Isolated lignin using the organosolv process contains small amounts of residual carbohydrates (1.24%), predominantly xylose (81.0%), from the degradation of hemicellulose. Organosolv not only achieved high lignin isolation yields of 70.8%, but also leads to sulfur-free (<0.05%) and high lignin purity (93.8%), clearly distinguishing it from industrial kraft lignin [16,21,61].

Comparisons of the chemical structures between lignin derived from organosolv and milled wood lignin (MWL) quantified the structural impact of the pretreatment. As highlighted in Table S1, Figures S1, S2, and S4, and detailed in previous investigations [60,62], organosolv promotes the depolymerization of the lignin macromolecular structure using β-O-4 acidolytic breakdown, also leading to a reduction in molecular weight. The lignin depolymerization is further supported by the observation of low recondensation on HSQC spectra, along with higher S/G ratios. The rise in phenolic hydroxyl content, as indicated by ^31^P NMR in Table S1, can also be attributed to lignin depolymerization, particularly with a significant increase in syringyl compared to guaiacyl groups, which are prominently represented within the aliphatic hydroxyls groups [62,63]. The modification of the LMP’s macromolecular structure through the organosolv isolation process, as shown previously, offers potential benefits for LNP manufacturing by altering lignin–solvent interactions. These modifications serve as the foundation for anti-solvent precipitation, which relies on polymer–solvent–antisolvent interactions, as demonstrated by prior studies on LNP production [43,45,46,55].

### 3.2. Exploration of 5 Different Experimental Parameters for the Antisolvent Precipitation Method (A)

Widely regarded as one of the most extensively studied methods for producing nanoparticles, the antisolvent precipitation offers several advantages, including simplicity, potential environmental friendliness (depending of solvents used), minimal equipment requirements, rapid processing, and high production yields [26,27]. However, drawbacks have been reported, such as potentially lower suspension concentrations compared to other techniques and a persistent lack of fine-tuning on final LNP characteristics due to the procedure itself [26]. The antisolvent method is based on the classical nucleation theory (CNT), which describes the spontaneous formation of auto-stabilized nano dispersions through homogeneous nucleation in a metastable region, with the nanoparticles themselves acting as surfactants [52]. As this phenomenon is based on polymer–solvent–antisolvent interactions (mainly solubility), it involves numerous parameters such as the polymer’s structure and concentration [43] and the properties of the solvents and antisolvents (type [49], proportions [52], mixing energy [64], flow rate [49], temperature, and pH [51]). The global mechanism is, therefore, challenging due to the complex physico-chemical interactions involved.

In a previous work [44], we analyzed the effect of the lignin polymer’s structure by varying the biomass source (hardwood, softwood, and herbaceous material), which affected the monomeric units (H, G, S) and the lignin isolation process with organosolv and kraft extractions. Lignin chemical structure variations affected the amphiphilic nature of lignin and its solubility in both solvents and antisolvents, changing the spontaneous precipitation process in the spinodal area during nucleation [44]. Additionally, other studies have focused on identifying solvents, in which lignin is the most soluble, such as THF, DMSO, and acetone [65], or ethanol [56], and their effects (concentration, proportion, temperature, or pH) on nanoparticle production [22]. However, there are still a lack of process parameters impact comparisons, as well as a reliable model allowing us to predict LNP size and manufacturing process conditions. This study aims to address the gaps by extensively investigating the synergistic effects of five synthesis parameters (lignin concentration, solvent flow rate, antisolvent composition, antisolvent ratio and antisolvent stirring speed). The detailed procedure is given in Section 2.4 and is illustrated in Figure 1. In a parametric study, each variable is studied sequentially while keeping the other parameters constant. The effect of the parameters on LNP properties (size distribution, homogeneity with polydispersity index PDI, stability with ζ-potential, and shape) is discussed in relation to the DLS and TEM results. Then, the range of the parameters’ values of interest is defined and used to perform a factorial design plan, allowing us to rank the order of the parameter’s influence as well as to define a correlation between LNP size and the most-influential parameters.

#### 3.2.1. Effect of Lignin Initial Concentration (x_1_)

**Figure 2:**
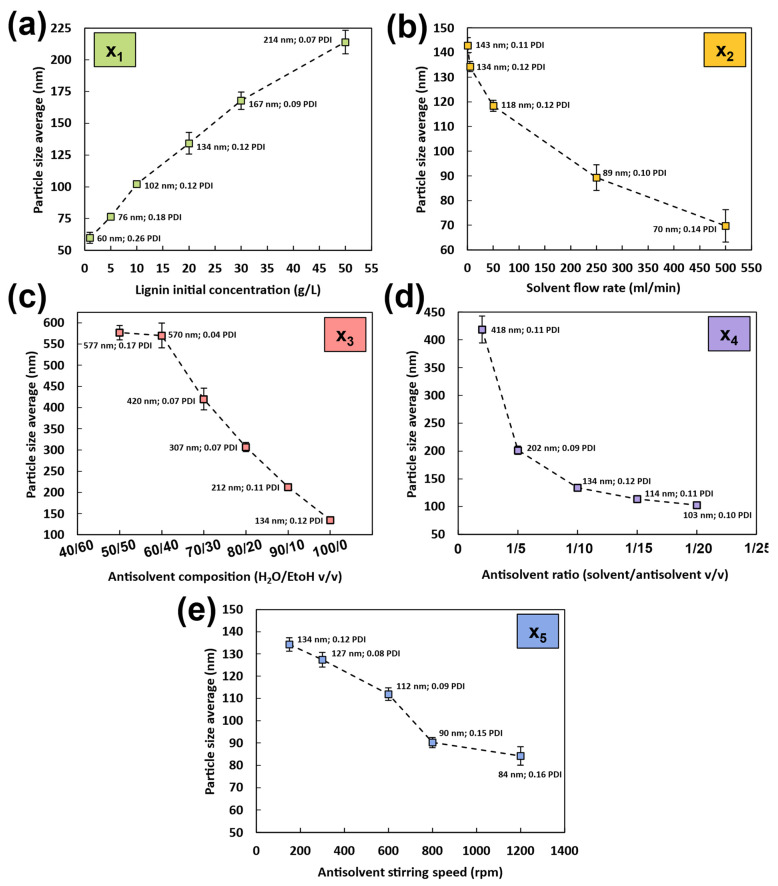
Graphs from DLS show the effect of the different parameters on LNP size and polydispersity index (PDI) with (**a**) x_1_ (initial lignin concentration, g/L), (**b**) x_2_ (solvent flow rate, ml/min), (**c**) x_3_ (antisolvent composition, water/EtOH, *v*/*v*), (**d**) x_4_ (antisolvent ratio, solvent/antisolvent, *v*/*v*), and (**e**) x_5_ (antisolvent stirring speed, rpm). The precise particle size distribution from DLS is given in Figure S5. Results of the different parameters on LNPs zeta potential are given in Figure S7.

**Figure 3:**
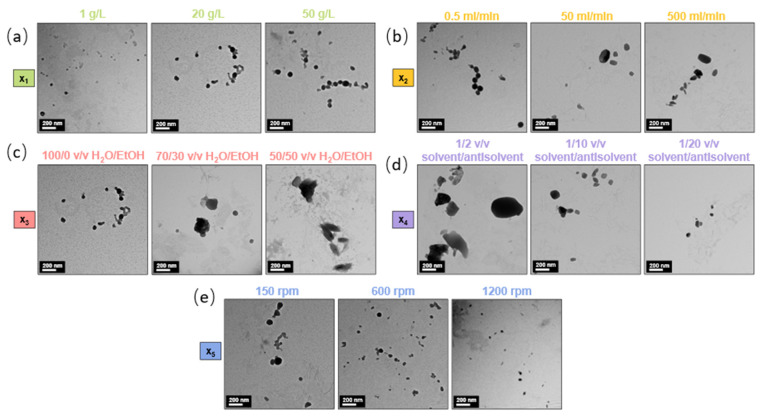
Photographs from the TEM show the effect of the different parameters on LNP morphology with (**a**) x_1_ (initial lignin concentration, g/L), (**b**) x_2_ (solvent flow rate, ml/min), (**c**) x_3_ (antisolvent composition, water/EtOH, *v*/*v*), (**d**) x_4_ (antisolvent ratio, solvent/antisolvent, *v*/*v*), (**e**), and x_5_ (antisolvent stirring speed, rpm). Precise parameters are given above the photographs. Scale bars for all photographs: 200 nm. Additional images for x_2_ and x_4_ are available in the Supplementary Materials.

The DLS data in Figure 2a and Figure S5a demonstrate that a higher initial lignin concentration in the solvent results in a significant increase in both average size and PDI of LNPs, thereby affecting the final properties of the produced nanoparticles. Similar results were widely observed in other studies [22,37,43,46,56,66]. From 1 g/L to 50 g/L, the particle size gradually increases from 60 to 214 nm with monomodal distributions (Figure S5a). At very low concentrations (i.e., 1 to 5 g/L), a broad distribution is observed, with particles from 20 to 300 nm (PDIs of 0.26 and 0.18 for 1 and 5 g/L concentrations). This can be attributed to the low number of particles produced, where the scattered intensity distribution is more influenced by the presence of a few large nanoparticles compared to higher concentrations, where the PDI is lower. TEM images in Figure 3a illustrate and reinforce the DLS results, indicating that high concentrations starting from 30 g/L led to particle aggregation and fusion during the nucleation process, negatively impacting the suspension stability. This is evidenced in Figure S7(x_1_) by a higher ζ-potential of −21.8 mV for 50 g/L compared to low concentrations (−27.9 mV and -26.4 mV for 1 and 5 g/L, respectively). The stability perturbation is accompanied by a change in morphology, with LNPs transitioning from individual spherical particles at concentrations from 1 to 20 g/L to more condensed, aggregated forms at higher concentrations (Figure 3a).

Thus, increasing the solvent concentration gradually increases the LNP size, decreases LNP stability by promoting the aggregation and fusion of particles, and changes the morphology during nucleation, which can even result in a bimodal distribution in certain cases, as observed by Manisekaran et al. [52].

#### 3.2.2. Effect of Solvent Flow Rate (x_2_)

As for other studies [37,50,51,56], the solvent flow rate parameter exhibited another important effect on LNP properties. From 0.5 to 500 mL/min flow rate, the particle size gradually decreases from 143 to 70 nm with monomodal distributions (Figure 2b and Figure S5b). According to Richter et al. [37], for flow rates > 400 mL/min, the size of the produced nanoparticles remained unchanged, indicating that this may be the threshold for maximum nuclei formation during the nucleation process. Regarding the stability and dispersity of the suspensions, the flow rate parameter does not seem to affect these properties significantly, as the particles exhibit similar PDI and ζ-potential values, ranging from 0.11 to 0.14 for PDI (Figure 2b) and from −28.4 to −24.5 mV for ζ-potential (Figure S7b). However, increasing the flow rate appears to change the morphology of the LNPs, as seen in Figure 3b, where nanoparticles shift from a spherical to an oval shape between 0.5 and 50 mL/min, and to flattened ovals at the highest flow rates. This morphological change may explain the slight variations in standard deviations between repetitions and the minor changes in stability observed.

The reason for these effects may be that a higher flow rate improves the mixing performance of the organic and aqueous phases, limiting the time available for aggregate growth during nucleation. Li et al. [50] explained that increasing the dropping speed restricts the time for aggregate growth, hindering the formation of a stable thermodynamic structure, resulting in a “frozen state”. Consequently, all single molecules contribute to nanoparticle formation once this “frozen state” is reached, leading to a low concentration in the solution and limiting the size growth of the particles over time.

#### 3.2.3. Effect of the Antisolvent Composition (x_3_)

As expected, significant changes were observed in DLS data (Figure 2c and Figure S5c) with varying ethanol concentrations in the antisolvent. An increase in ethanol content leads to a corresponding increase in particle size, ranging from 577 nm for a 50/50 water/ethanol ratio to 134 nm for the 100/0 water/ethanol ratio. As previously explained, the antisolvent precipitation method relies on the solubility interaction of the polymer with both the solvent and the antisolvent. When the antisolvent contains ethanol, in which lignin is soluble, nucleation remains incomplete. This results in a mixed solution/suspension rather than a fully formed suspension, as the lignin does not fully precipitate into nanoparticles. This effect is clearly observed in the TEM images in Figure 3c, where the suspension of stable spherical nanoparticles in a 100/0 water/ethanol antisolvent composition transitions into partially solubilized particles with irregular forms when the ethanol ratio increases. The increase in ethanol content in the antisolvent results in a lignin solution/suspension rather than a pure suspension. This change in the nature of the suspension raises concerns about the accuracy of the nanoparticle properties analyzed using DLS. Notably, the ζ-potential in Figure S7c decreases significantly, from −24.5 mV for the 100/0 water/ethanol mixture to −7.1 mV for the 50/50 water/ethanol mixture, with an immediate drop to −19.1 mV at a 90/10 water/ethanol ratio. Additionally, with size distributions approaching the micrometer range (Figure S5c), the DLS detection limits are reached, which also affects the reliability of the PDI results.

#### 3.2.4. Effect of the Antisolvent Volume (x_4_)

As observed with previous parameters, the DLS data in Figure 2d and Figure S5d indicate that increasing the antisolvent volume (water ratio) significantly reduces the average particle size and distribution, from 418 nm for a 1/2 *v*/*v* solvent/antisolvent ratio to 103 nm for a 1/20 *v*/*v* solvent/antisolvent ratio. The particle size distributions in Figure S5d remain monomodal, indicating consistent nanoparticle formation. These findings align with previous work by Ju et al. [49]. The transition from a 1/2 to a 1/5 ratio notably impacts particle size, decreasing it from 418 to 202 nm, and at higher antisolvent volumes it approaches a limit value (114 nm for 1/15 and 103 nm for 1/20 ratios). The PDI varies minimally, maintaining low values between 0.09 and 0.11, which confirms the monomodal distribution across all antisolvent volumes. Figure S5d also shows that increasing the antisolvent water volume significantly enhances the stability of the suspensions, as evidenced by a slight reduction in ζ-potential from −12.1 mV at 1/2 *v*/*v* to −29.5 mV at 1/15 *v/v*, respectively. As with other parameters, larger LNPs typically exhibit lower stability and ζ-potential values. The increased antisolvent volume reduces the final lignin concentration, acting counter to the effect of the initial lignin concentration. TEM images from Figure 3d corroborate the DLS results, highlighting the significant impact of antisolvent volume on particle size and revealing the high standard deviation associated with the 1/2 *v*/*v* suspension. Like the initial lignin concentration parameter, low dilution ratios (i.e., high concentrations) lead to particle aggregation and fusion during nucleation, negatively impacting the particle’s morphology and suspension stability. As explained by Tan et al. [67], different solvent/antisolvent ratios create varying levels of supersaturation, which control LNP growth [68]. A high antisolvent volume induces a high supersaturation level, increasing the nucleation rate and effectively controlling nanoparticle growth.

#### 3.2.5. Effect of the Antisolvent Stirring Speed (x_5_)

The DLS data in Figure 2e and Figure S5e, consistent with the work of Xiong et al. [51], show that increasing the stirring speed gradually decreases the average LNP size from 134 nm at 150 rpm to 84 nm at 1200 rpm. Like the effect of the solvent flow rate, higher stirring speeds result in monomodal distributions by improving the mixing performance of the organic and aqueous phases, thereby limiting the time available for aggregate growth during nucleation. Additionally, there appears to be a threshold at around 800 rpm, beyond which the nanoparticle’s size remains unchanged, suggesting a limit for maximum nuclei formation during the nucleation process. The PDI shows only minor variation, increasing slightly from 0.08 at 300 rpm to 0.12 at 1200 rpm, leading to a conclusion like that for the solvent flow rate parameter. Figure S7e indicates that increasing the stirring speed has no significant impact on particle stability, with ζ-potential values ranging from −24.5 mV at 150 rpm to −25.6 mV at 1200 rpm. However, as seen in Figure S3(x_5_), the stirring speed appears to influence the morphology of the LNPs, shifting from spherical LNPs at 150 rpm to small, flattened ovals at 1200 rpm.

### 3.3. Lignin Nanoparticle Prediction Model Using Experimental Design (B)

The previous parametric study leads to detailed knowledge of the influence of each process parameters taken separately on LNP characteristics. However, the relative impact of the parameters when varying simultaneously is needed to complete the understanding of the process and to evaluate its ability to be transferred in a production environment. To be able to produce LNPs with desirable properties such as particle size and particle size distribution, high stability over time, high concentration, simplicity of the production process, minimal solvent usage, and low energy consumption, the impacts of the following parameters have been studied by varying all of them simultaneously in a range of values defined according to the results of the parametric study:

- x_1_ (initial lignin concentration, g/L): 20 g/L, to prevent aggregation and particle fusion during the nucleation process.
- x_2_ (solvent flow rate, ml/min): 5 mL/min, to ensure interesting LNP properties while having a low energy consumption.
- x_3_ (antisolvent composition, H_2_O/EtOH, *v*/*v*): 100% H_2_O, to ensure the correct nucleation process and produce authentic LNP suspensions while reducing EtOH consumption.
- x_4_ (antisolvent ratio, solvent/antisolvent, *v*/*v*): 1/10, to fine-tune lignin concentration and nucleation while reducing H_2_O use.
- x_5_ (antisolvent stirring speed, rpm): 150 rpm, combined with the solvent flow rate, enhances mixing and supersaturation, thereby improving nucleation. This value also optimizes the LNPs properties while maintaining low energy consumption.

The Fractional Factorial Design (FFD) used to evaluate the impact of each parameter and to produce a predictive model for LNP synthesis using antisolvent precipitation is given in Section 2.4, and the factors studied are listed in Figure 1B. Table 2 shows the factor arrangements, the list of randomly ordered runs, and the response values obtained for each experiment of the experimental set created using Minitab software. The response in Table 2 is the average LNP size (detailed size distribution from DLS are available in Figure S8). Table S2 presents the ζ-potential response to the suspensions produced according to the FFD parameters.

The narrow range of values used for x_1_ and x_2_ has been defined to keep the liquid to solid phase change mechanism during LNP formation unchanged, considering that the lignin phase diagram is not known. This is an important prerequisite to rigorously characterize the impact of each factor on the nanoparticle building mechanism.

In that frame, according to Figures S9–S11, the most significant factors of the model are x_3_ and x_4_ as independent factors, followed by the quadratic effect of x_3_–x_4_. This aligns well with the results in Figure 2. The LNP’s size and size distribution is therefore mostly influenced by the antisolvent composition and ratio (x_3_, x_4_), while the initial lignin concentration (x_1_), antisolvent stirring speed (x_5_), and solvent flow rate (x_2_) appeared to be less relevant and quite ineffective in controlling LNP size. As shown by the variance analysis, a linear first order correlation to predict LNP size as a function of manufacturing conditions can be written as follows:

y=0.798x+28.184

**Figure 4:**
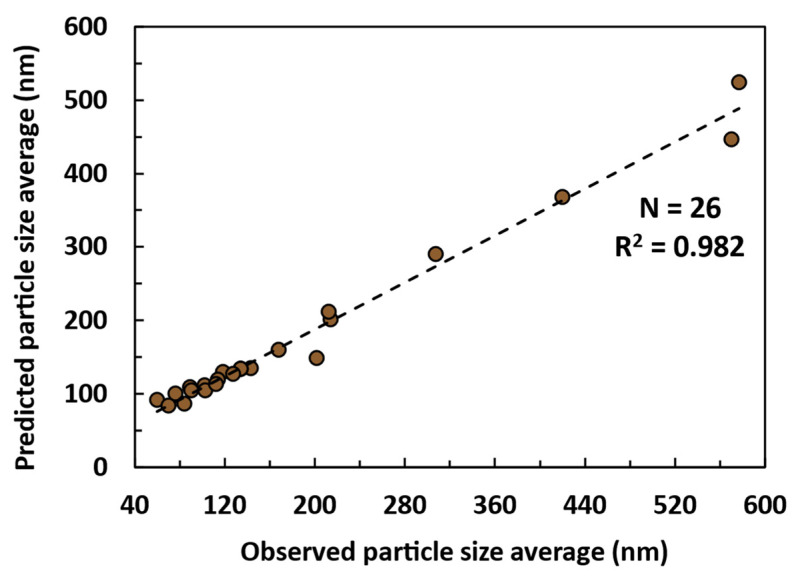
Correlation between the predictive model and 26 experiments from the experimental design with the associated response (LNP size average, nm). The model considers the following factors and their alias: x_1_ (initial lignin concentration, g/L), x_2_ (solvent flow rate, ml/min), x_3_ (antisolvent composition, water/EtOH, *v*/*v*), x_4_ (antisolvent ratio, solvent/antisolvent, *v*/*v*), and x_5_ (antisolvent stirring speed, rpm).

In addition, the relevance of the model has been demonstrated thanks to the correlation between the predicted versus observed responses for each of the 26 (N value) experiments calculated using the model (Figure 4). This linear representation displays a correlation coefficient of R^2^ = 0.982 and a model validity of 97.9%. The linear model is based on 2 independent factors, and an interaction factor provides essential information to design and implement the manufacturing of lignin nanoparticles at production scale in an industrial environment.

## 4. Conclusions

This study extensively explores the antisolvent precipitation method for producing LNPs, which is essential for the future of biorefineries and large-scale lignin valorization. For several years, research has focused on transitioning to the nanoscale to enhance the intrinsic properties of polymers. However, in the case of lignin, current applications are primarily hindered by the highly condensed and sulfur-rich chemical structure of kraft lignin, as well as by existing nanoscale reduction processes that are energy-consuming, lack control and repeatability, and rely on toxic solvents such as THF and DMSO.

Here, we generate a pure lignin from beech biomass residues using a semi-industrial organosolv process, which is chemically ideal for the fabrication of nanoparticles, as shown by Girard et al. [44]. By studying five different parameters of the antisolvent precipitation method with ethanol and water for the first time, this work demonstrates that the antisolvent composition and solvent/antisolvent ratio are the most crucial factors for controlling the nucleation phenomenon according to the classical nucleation theory. Starting from an average size of 577 nm, the LNPs were reduced to 134 nm by shifting the antisolvent composition from 50/50 *v*/*v* water/ethanol to 100/0 *v*/*v* water/ethanol. Additionally, increasing the solvent/antisolvent ratio from 1/2 to 1/20 reduced the average LNP size by 315 nm. The proposed optimization of these different parameters in Section 3.3 led to an improvement in PDI, ζ-potential, and particle morphology, resulting in tailored environmentally friendly suspensions with desired properties. Additionally, by conducting these experiments within an experimental design framework, the former hypothesis regarding the most important factors was demonstrated and an accurate predictive model for LNP size properties was developed. With a linear correlation coefficient of R^2^ = 0.982 and a model validity of 97.9%, the anti-solvent precipitation model presents essential information to implement the process towards production scale.

This study describes a globally controlled top-down process using biomass residues to produce tailored lignin nanoparticles with enhanced properties and reduced environmental impact. The process allows for the optimization and prediction of LNP characteristics for industrial applications, paving the way for future developments.

## Figures and Tables

**Table 1:** 1—Chemical composition of the raw biomass used in this study (results were taken from Girard et al. work [44]). 2—Main data for lignin isolation from 10 L organosolv process. Extraction repetitions were performed from same dry biomass with equivalent granulometry. The initial particle diameter was 8 mm, and no acid catalysis was used. Cellulose, hemicellulose, and lignin contents were based on the solid residue analysis. Lignin macroparticles (LMPs) extraction yields (wtc %) are based on raw lignin biomass content. Each value is the mean of three repetitions.

1. Raw Material (%)	Cellulose	Hemicellulose	Lignin	Extractives	Ashes	Total
	47.8 ± 1.5	22.5 ± 0.9	23.7 ± 0.2	2.7 ± 0.3	0.7 ± 0	97.4 ± 2.9
2. Organosolv solid residue (%)	Cellulose	Hemicellulose	Lignin	Mass loss yield (wt %)	LMPs purity (%)	LMPs isolation yields (wtc %)
	51.5 ± 1.8	13.0 ± 0.7	13.0 ± 0.4	46.0 ± 0.1	93.8 ± 0.3	70.8 ± 0.2

**Table 2:** The experimental design summary shows the different factors, runs, and the associated response (LNP size average, nm). The factors are x_1_ (initial lignin concentration, g/L), x_2_ (solvent flow rate, ml/min), x_3_ (antisolvent composition, water/EtOH, *v*/*v*), x_4_ (antisolvent ratio, solvent/antisolvent, *v*/*v*), and x_5_ (antisolvent stirring speed, rpm).

Run	Design Factors (2^5−1^)					Design Response
	x_1_	x_2_	x_3_	x_4_	x_5_	LNPs Size Average
1	10	100	80	5	150	323 ± 6
2	20	2	100	5	1000	112 ± 3
3	10	100	80	20	1000	109 ± 2
4	20	2	100	20	150	114 ± 4
5	20	100	80	5	1000	266 ± 2
6	10	2	100	5	150	128 ± 3
7	20	100	80	20	150	173 ± 3
8	10	2	100	20	1000	50 ± 2
9	20	2	80	5	150	340 ± 6
10	10	100	100	5	1000	87 ± 3
11	20	2	80	20	1000	127 ± 3
12	10	100	100	20	150	62 ± 2
13	10	2	80	5	1000	237 ± 5
14	20	100	100	5	150	134 ± 3
15	10	2	80	20	150	153 ± 3
16	20	100	100	20	1000	73 ± 2
17	15	51	90	12.5	575	132 ± 2

## Data Availability

The original contributions presented in the study are included in the article/Supplementary Materials, further inquiries can be directed to the corresponding author/s.

## Supplementary Materials

The following supporting information can be downloaded at: https://www.mdpi.com/article/10.3390/nano14221786/s1, Experimental Procedure: Beech milled wood lignin extraction isolation procedure. Lignin Molecular Weight Characterization by SEC. Lignin Structure Analysis by HSQC NMR. Lignin Hydroxyl Group Content by 31P NMR Analysis. Table S1: LMPs characterization from organosolv and MWLs. Figure S1: Size Exclusion Chromatography results with molecular weight distributions curves for organosolv and MWLs. Figure S2: Identification of Primary Lignin 13C-1H Cross-Peaks in HSQC NMR. Figure S3: HSQC NMR spectra’s for organosolv and MWLs. Figure S4: Quantitative 31P NMR spectra for organosolv isolated lignin. Figure S5: Particle size distribution from DLS according to the different parameters used for LNPs synthesis. Figure S6: Additional photographs from the TEM. Figure S7: Graphs from DLS show the effect of the different parameters on LNPs zeta potential. Table S2: The experimental design summary shows the different factors, runs and the associated response (ζ-potential in mV). Figure S8: Particle size distribution in terms of relative scattered intensity from DLS according to the experimental design runs. Figure S9: Summary of the experimental design results. Figure S10: The experimental design results concerning the different factors. Figure S11: The main effects plot for the response (particle size average, nm). Refs. [44,57,69,70] are cited in the Supplementary Materials file.
