# Supplementary material for: Modeling the Production Process of Lignin Nanoparticles Through Anti-Solvent Precipitation for Properties Prediction
Source: Nanomaterials (Basel). 2024 Nov 6;14(22):1786. doi: 10.3390/nano14221786 (PMC11597156; doi:10.3390/nano14221786)
Supplement: Supplementary file 1 [file nanomaterials-14-01786-s001.zip › nanomaterials-3266279-supplementary.pdf]

Supplementary materials for the article:

Article

Modeling the Production Process of Lignin Nanoparticles Through Anti-Solvent Precipitation for Properties Prediction

Victor Girard [1,*], Laurent Marchal-Heussler [2], Hubert Chapuis [1], Nicolas Brosse [1], Nadia Canilho [3] and Isabelle Ziegler-Devin [1,*]

[1] LERMAB, Faculty of Science and Technology, University of Lorraine, INRAe, F-54000 Nancy, France;
hubert.chapuis@univ-lorraine.fr (H.C.); nicolas.brosse@univ-lorraine.fr (N.B.);
[2] ENSIC, University of Lorraine, F-54000 Nancy, France; laurent.marchal-heussler@univ-lorraine.fr
[3] L2CM, Faculty of Science and Technology, University of Lorraine, CNRS, F-54000 Nancy, France;
nadia.canilho@univ-lorraine.fr
* Correspondence: victor.girard@univ-lorraine.fr (V.G.); isabelle.ziegler@univ-lorraine.fr (I.Z.-D.)

Experimental Procedure

Beech milled wood lignin (MWL) isolation procedure from the Qian et al. [1] work.

The beech sawdust was extensively milled by the ball milling rotary (PM100, Retsch, Germany) and sifted with a 0.05 mm sieve. The milled powder (100 g) was extracted with a Soxhlet extraction apparatus by toluene/ethanol (2/1 by vol) for 6 h. The free extractive powder was then mixed with dioxane/water solution (96/4 by vol) and then stirred in the dark for 24 h with a liquid/solid ratio of 10/1, followed by filtration. The procedure was repeated three times. The combined dioxane/water extract was concentrated by rotary evaporation and then lyophilized for 48 h. The lignin extract was dissolved in 5 mL of acetic acid (90%) for 1 h at room temperature and was then added to distilled water (80 mL/g lignin). After several centrifugations, the solid phase was dried for 24 h at 40 °C, and then it was dissolved in 10 mL of 1,2-dichloroethane/ethanol mixture (2/1, by vol). The mixture was then centrifuged to remove the insoluble solid; the liquid phase was added into 200 mL of diethyl ether. After centrifugation, the lignin extract was washed three times with diethyl ether and dried for 48 h at 40 °C. The MWL isolation yields were 1.81 wt%.

Lignin Molecular Weight Characterisation by SEC from the work of Girard et al. [2].

Size exclusion chromatography (SEC) was used to determine the molecular weight distributions and average molecular weights of LMP fractions. Initially, each lignin sample underwent dissolution in NaOH (10 mM, 5 mg/mL) with magnetic agitation for 24 hours, followed by filtration through 0.45 µm PTFE filters. The choice of NaOH as a solvent, instead of the conventional tetrahydrofuran (THF), enhanced data precision when employing pullulans as standards [3]. A Shimadzu Prominence chromatography system, comprising a Shimadzu SPD-20A UV detector (280 nm and 254 nm), a refractive index detector (RID, Shimadzu RID-20A), and a combination of Phenomenex PolySep-SEC GFC-P 2000 and PolySep-SEC GFC-P 3000 columns, was employed for the analyses. The separation was conducted at 35°C with elution using NaOH (10 mM) at a flow rate of 0.4 mL/min. Subsequently, the calibration curve was generated using Agilent Technologies GPC/SEC calibration kits for pullulan standards (Agilent PL2090-0101), following the Steinmetz et al. [4] method.

Lignin Structure Analysis by HSQC NMR from the work of Girard et al. [2].

The lignin structure was analysed by heteronuclear single quantum coherence (HSQC) nuclear magnetic resonance (NMR) spectroscopy. In brief, 100 mg of purified and dried LMPs was dissolved in 500 µL of dimethysulfoxide-d6 (DMSO-d6 99.8%) for the 13C–1 H HSQC analysis. Spectra were acquired employing the Bruker Avance III 400 MHz spectrometer at 50 °C, with a relaxation delay of 25 s.

Lignin Hydroxyl Group Content by 31P NMR Analysis from the work of Girard et al. [2].

The lignin hydroxyl group content was determined by phosphorus-31 NMR spectroscopy. Briefly, 25 mg of purified and dried LMPs was dissolved in 400 µL of a mixed solution (A) of pyridine/deuterated chloroform (1.6/1, v/v) in a 2 mL vial. Then, 150 µL of other mixed solutions of chromium (III) acetylacetonate 97% (3.6 mg/mL of A) and cyclohexanol (4.0 mg/mL of A) was added, respectively, as the relaxation reagent and internal standard. Then, the solution was derivatized with 50 µL of 2-chloro-4,4,5,5-tetramethyl-1,3,2-dioxa-phospholane (TMDP) before being vortexed and analysed on the Bruker Avance III HD 300 MHz spectrometer at 25 °C with relaxation delay of 2 s. All NMR data were processed by using Topspin 4.1.0 software (Bruker Bio Spin).

Table S1: LMPs characterisation from organosolv and MWL lignin's. Results including average molecular weight from SEC, quantification of side chains and aromatic regions from HSQC NMR, and evaluation of functional groups from ^{31}P NMR. [a] Results of ^{31}P NMR for milled wood lignin were taken from Qian et al. [1] work.

**Average M_w (kDa)**	Organosolv	MWL
M_w	17.8	65.7
**Linkages (%)**	Organosolv	MWL
β–O–4	31.3	47.9
β–5	4.8	3.9
β–β	8.9	12.0
S/G	2.0	0.6
**Hydroxyl group (mmol.g^{-1})**	Organosolv	MWL[a]
Aliphatic (R) –OH	3.14	2.52
Phenolic –OH	2.14	0.48
Syringyl	1.12	0.09
Guaiacyl	0.89	0.33
p-Hydroxyphenyl	0.13	0.03
COOH	0.00	0.03
Total –OH	5.28	3.03
PhOH/ROH	0.68	0.19
S/G –OH	1.25	0.27

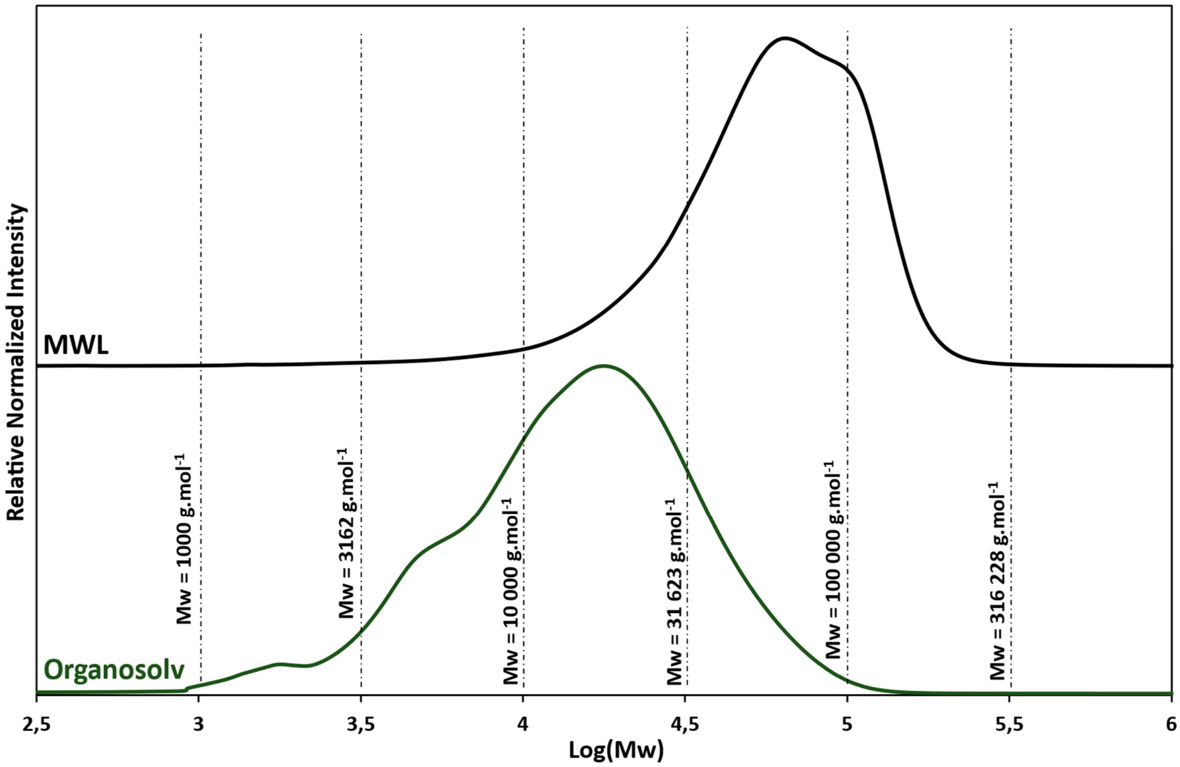

Figure S1: Size Exclusion Chromatography results with molecular weight distributions curves for organosolv and MWL lignin's with relative intensity normalized to 1. Milled wood lignin is shown in black and organosolv isolated lignin in dark green.

δ_C/δ_H (ppm)	Label	Assignement
31.3/2.5	A_{ek}	C_β-H_β in Aryl ethyl ketones end groups
34.4/1.7	A_{prop}	C_α-H_α in Aryl propanols end groups
39.5/2.4	A_{ac}	C_α-H_α in Arylacetic acid end groups
42.3/1.9	$S_{resinol}$	C_β-H_β in Secoisolariciresinols substructures
53.6/3.45	B_β	C_β-H_β in phenylcoumaran β-5′ substructures (B)
54.0/3.04	C_β	C_β-H_β in resinol substructures β-β′ (C)
55.6/3.73	OMe	C-H in methoxyls
59.7/2.77	D_β	C_β-H_β in spirodienone substructures (D)
60/3.10-4.00	A_γ	C_γ-H_γ in β-O-4' substructures (A)
61.4/4.10	I_γ	C_γ-H_γ in p-hydroxycinnamyl alcohol end groups (I)
63/3.19	X_5	C_5-H_5 in β-D-xylopyranoside (X)
63.2/4.33-4.49	A'_γ	C_γ-H_γ in γ-acylated β-O-4' substructures (A' and A")
63.3/3.75	B_γ	C_γ-H_γ in phenylcoumaran β-5′ substructures (B)
71.0/3.82 and 4.18	C_γ	C_γ-H_γ in resinol substructures β-β′ (C)
71.8-4.86	A,A'_α	C_α-H_α in β-O-4' substructures (A) and γ-acylated β-O-4' substructures (A')
73.1/3.03	X_2	C_2-H_2 in β-D-xylopyranoside (X)
74.1/4.50	X'_2	C_2-H_2 in 2-O-acetyl-β-D-xylopyranoside (X)
74.7/3.22	X_3	C_3-H_3 in β-D-xylopyranoside (X)
75.8/4.87	X'_3	C_3-H_3 in 3-O-acetyl-β-D-xylopyranoside (X)
76.6/3.49	X_4	C_4-H_4 in β-D-xylopyranoside (X)
79.5/4.12	$D_{\beta'}$	$C_{\beta'}$-$H_{\beta'}$ in spirodienone substructures (D)
81.1/3.10	U_4	C_4-H_4 in 4-O-methyl-α-D-GlcUA
81.2/5.07	D_α	C_α-H_α in spirodienone substructures (D)
81.8/4.76	BE_α	C_α-H_α in benzyl ether substructures
82.7/4.55	$A_{\beta(H)}$	C_β-H_β in β-O-4′ substructures linked to a H unit (A)
83.7/5.21	F_β	C_β-H_β in α-oxidized (C_α=O) β-O-4′ substructures (F)
84.3/4.29	$A_{\beta(G)}$	C_β-H_β in β-O-4′ substructures linked to a G unit (A)
85.6/4.66	C_α	C_α-H_α in resinol β-β′ substructures (C)
85.9/3.84	E_β	C_β-H_β in dibenzodioxocin substructures (E)
87.1/4.04	$A_{\beta(S)}$	C_β-H_β in β-O-4′ substructures linked to a S unit (A)
87.9/5.50	B_α	C_α-H_α in phenylcoumaran β-5′ substructures (B)
103.8/6.71	$S_{2,6}$	$C_{2,6}$-$H_{2,6}$ in etherified syringyl units (S)
106.2/7.07 and 7.23	$S'_{2,6}$	$C_{2,6}$-$H_{2,6}$ in oxidized (C_α=O) syringyl units (S')
110.9/6.98	G_2	C_2-H_2 in guaiacyl units (G)
111.4/7.51	G'_2	C_2-H_2 in oxidized (C_α=O) guaiacyl units (G')
111.6/7.31	CA_2	C_2-H_2 in coniferaldehyde
113.3/6.22	$D_{2'}$	$C_{2'}$-$H_{2'}$ in spirodienone substructures (D)
114.9/6.77	G_5	C_2-H_2 in guaiacyl units (G)
118.9/6.07	$D_{6'}$	$C_{6'}$-$H_{6'}$ in spirodienone substructures (D)
119.0/6.80	G_6	C_6-H_6 in guaiacyl units (G)
123.3/7.60	G'_6	C_6-H_6 in oxidized (C_α=O) guaiacyl units (G')
126.1/6.76	J_β	C_β-H_β in cinnamaldehyde end groups (J)
127.9/6.90	$H_{2,6}$	$C_{2,6}$-$H_{2,6}$ in p-hydroxyphenyl units (H)
128.2/6.25	I_β	C_β-H_β in p-hydroxycinnamyl alcohol end groups (I)
128.4/6.44	I_α	C_α-H_α in p-hydroxycinnamyl alcohol end groups (I)
131.2/7.67	$PB_{2,6}$	$C_{2,6}$-$H_{2,6}$ in p-hydroxybenzoate substructures (PB)

Figure S2: Identification of Primary Lignin 13C-1H Cross-Peaks in HSQC NMR.

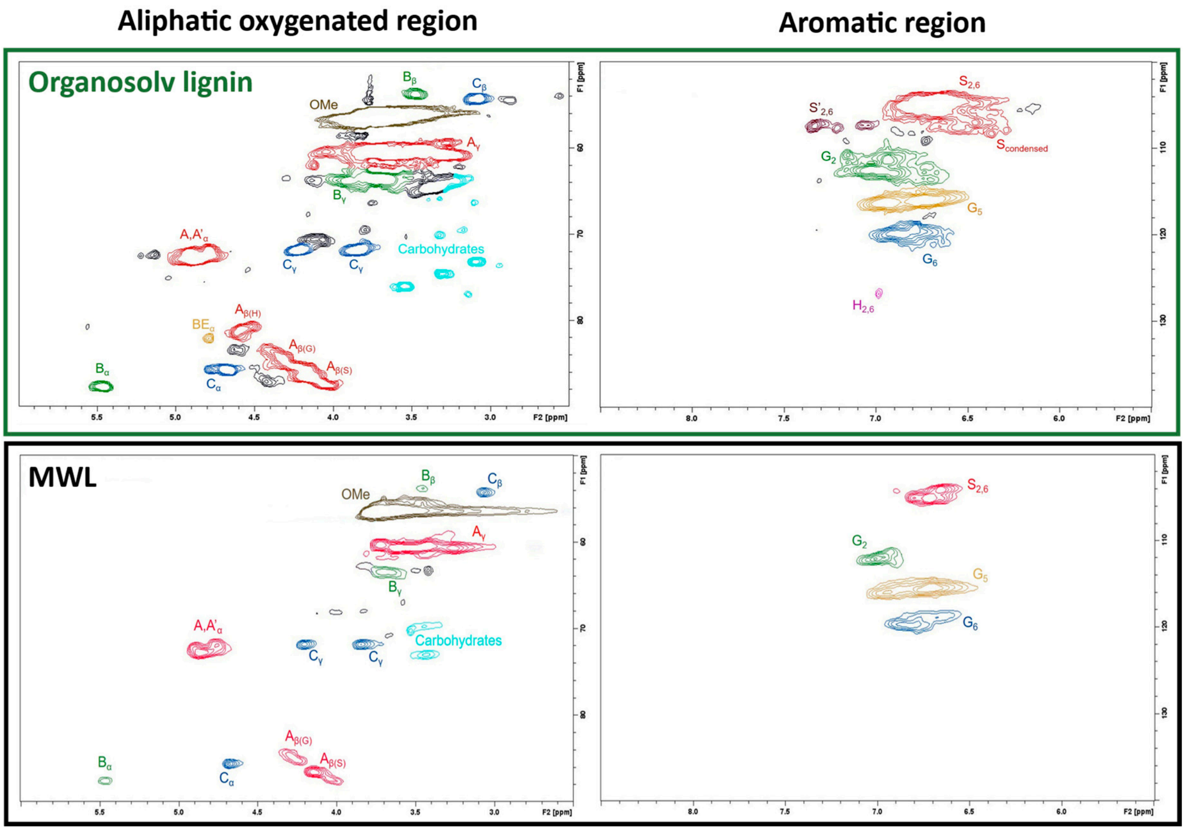

Figure S3: HSQC NMR spectra's for organosolv and MWL lignin's. Milled wood lignin is shown in black (at the bottom) and organosolv isolated lignin in dark green (at the top).

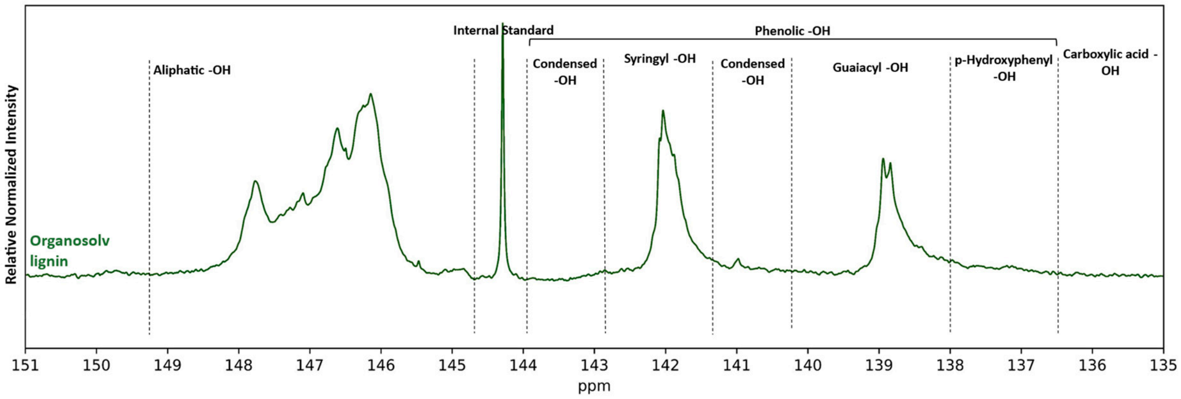

Figure S4: Quantitative 31P NMR spectra for organosolv isolated lignin.

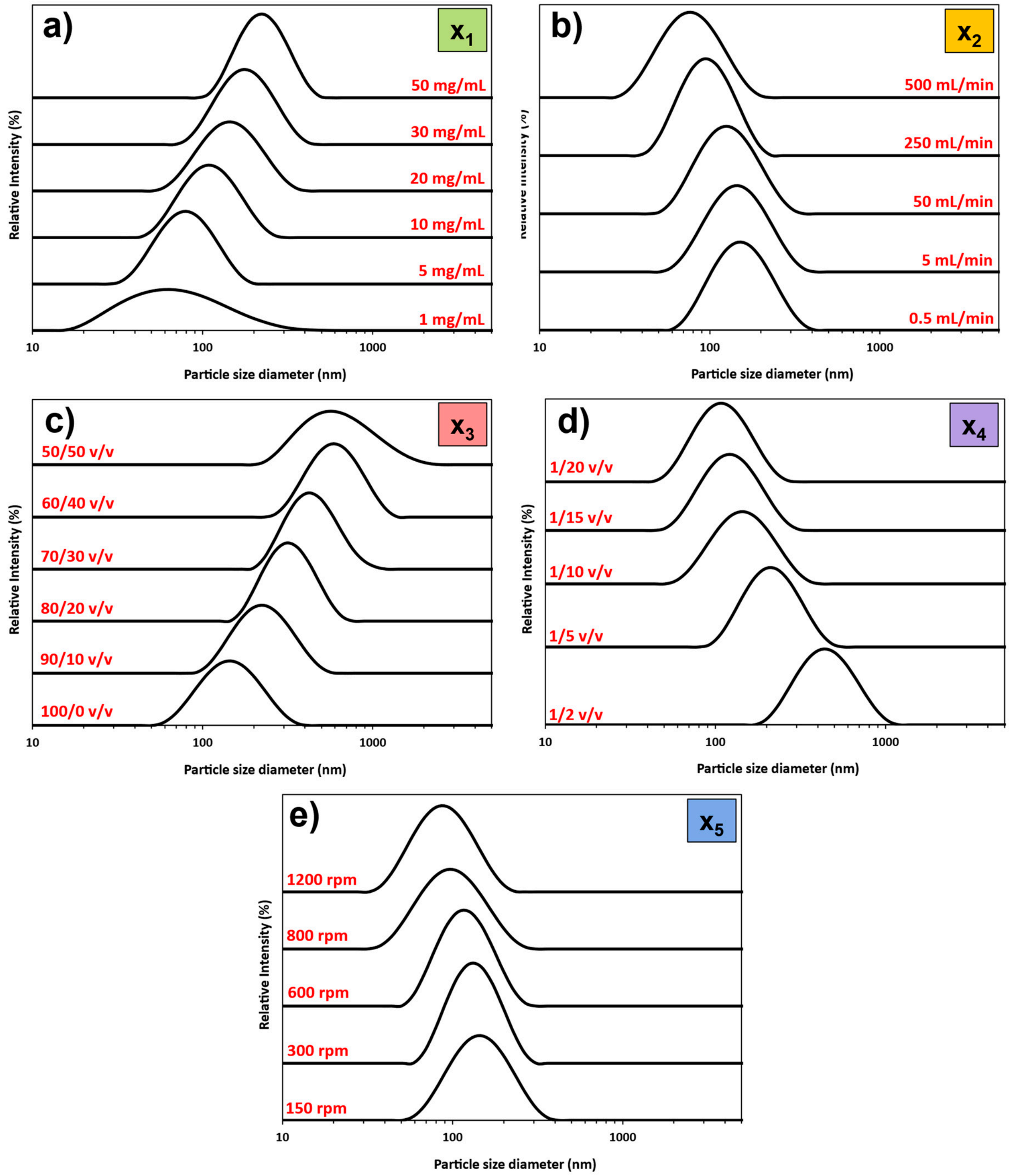

Figure S5: Particle size distribution in terms of relative scattered intensity from DLS according to the different parameters used for LNPs synthesis. x_i represents the different variables (values in bold brackets) with a) x_1 (initial lignin concentration, g/L), b) x_2 (solvent flow rate, ml/min), c) x_3 (antisolvent composition, water/EtOH, v/v), d) x_4 (antisolvent ratio, solvent/antisolvent, v/v), and e) x_5 (antisolvent stirring speed, rpm).

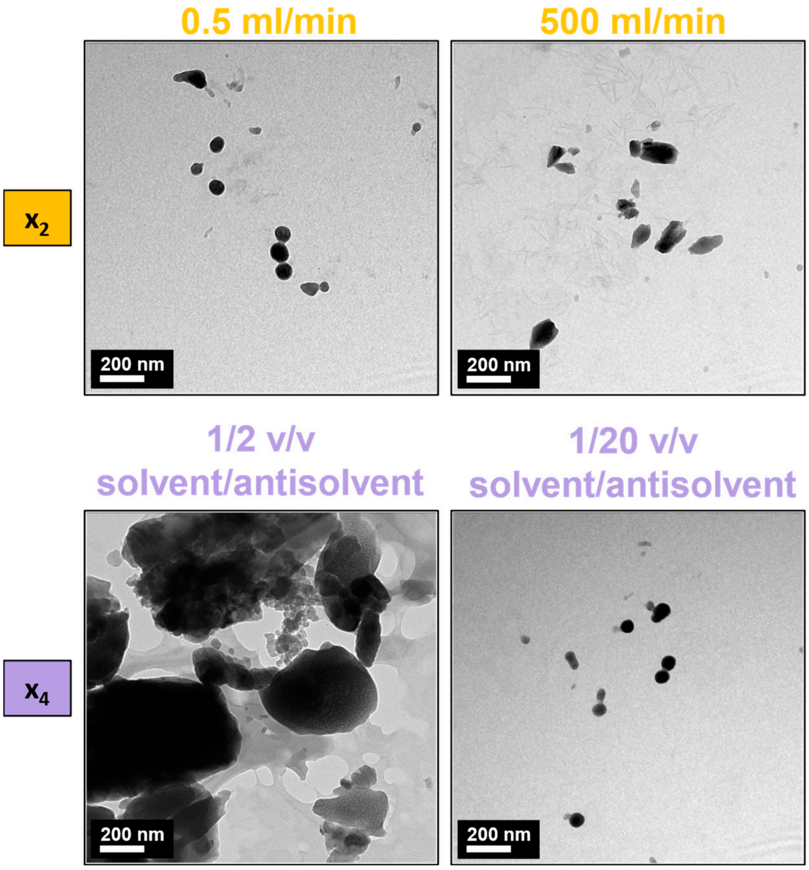

Figure S6: Additional photographs from the TEM microscopy for x_2 (solvent flow rate, ml/min) and x_4 (antisolvent ratio, solvent/antisolvent, v/v). Precise parameters are given above the photographs. Scale bars for all photographs: 200 nm.

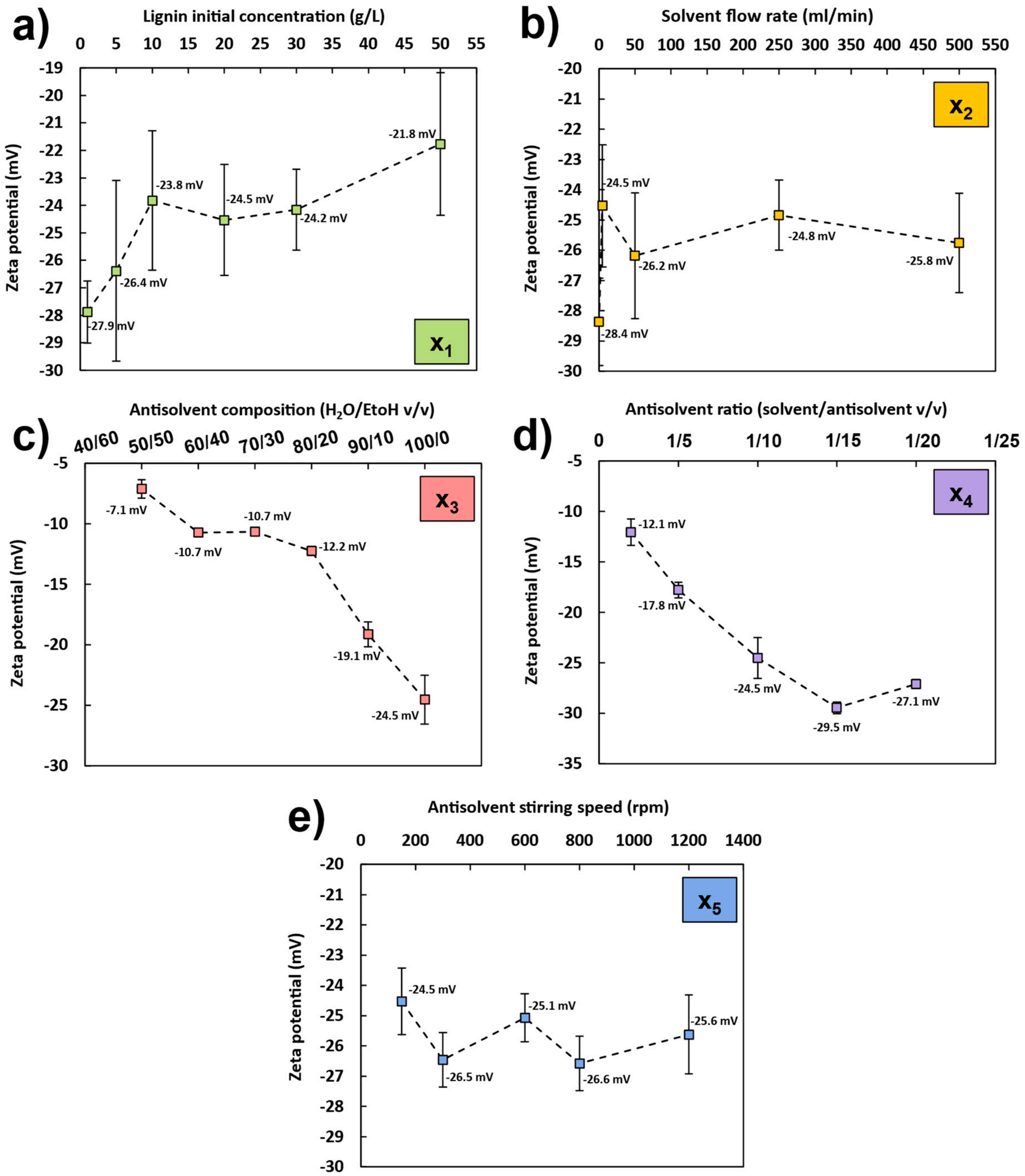

Figure S7: Graphs from DLS show the effect of the different parameters on LNPs ζ-potential with a) x_1 (initial lignin concentration, g/L), b) x_2 (solvent flow rate, ml/min), c) x_3 (antisolvent composition, water/EtOH, v/v), d) x_4 (antisolvent ratio, solvent/antisolvent, v/v), and e) x_5 (antisolvent stirring speed, rpm).

Table S2: The experimental design summary shows the different factors, runs and the associated response (ζ-potential in mV). The factors are x_1 (initial lignin concentration, g/L), x_2 (solvent flow rate, ml/min), x_3 (antisolvent composition, water/EtOH, v/v), x_4 (antisolvent ratio, solvent/antisolvent, v/v), and x_5 (antisolvent stirring speed, rpm). The final run is the central point.

**Run**	**Design Factors (2^{5-1})**					**Design response**
	x_1	x_2	x_3	x_4	x_5	ζ-potential (mV)
1	10	100	80	5	150	**-12.0**
2	20	2	100	5	1000	**-21.0**
3	10	100	80	20	1000	**-16.8**
4	20	2	100	20	150	**-35.5**
5	20	100	80	5	1000	**-14.9**
6	10	2	100	5	150	**-18.4**
7	20	100	80	20	150	**-18.3**
8	10	2	100	20	1000	**-29.3**
9	20	2	80	5	150	**-13.1**
10	10	100	100	5	1000	**-16.4**
11	20	2	80	20	1000	**-19.7**
12	10	100	100	20	150	**-30.3**
13	10	2	80	5	1000	**-12.4**
14	20	100	100	5	150	**-24.6**
15	10	2	80	20	150	**-16.4**
16	20	100	100	20	1000	**-35.8**
17	15	51	90	12.5	575	**-22.9**

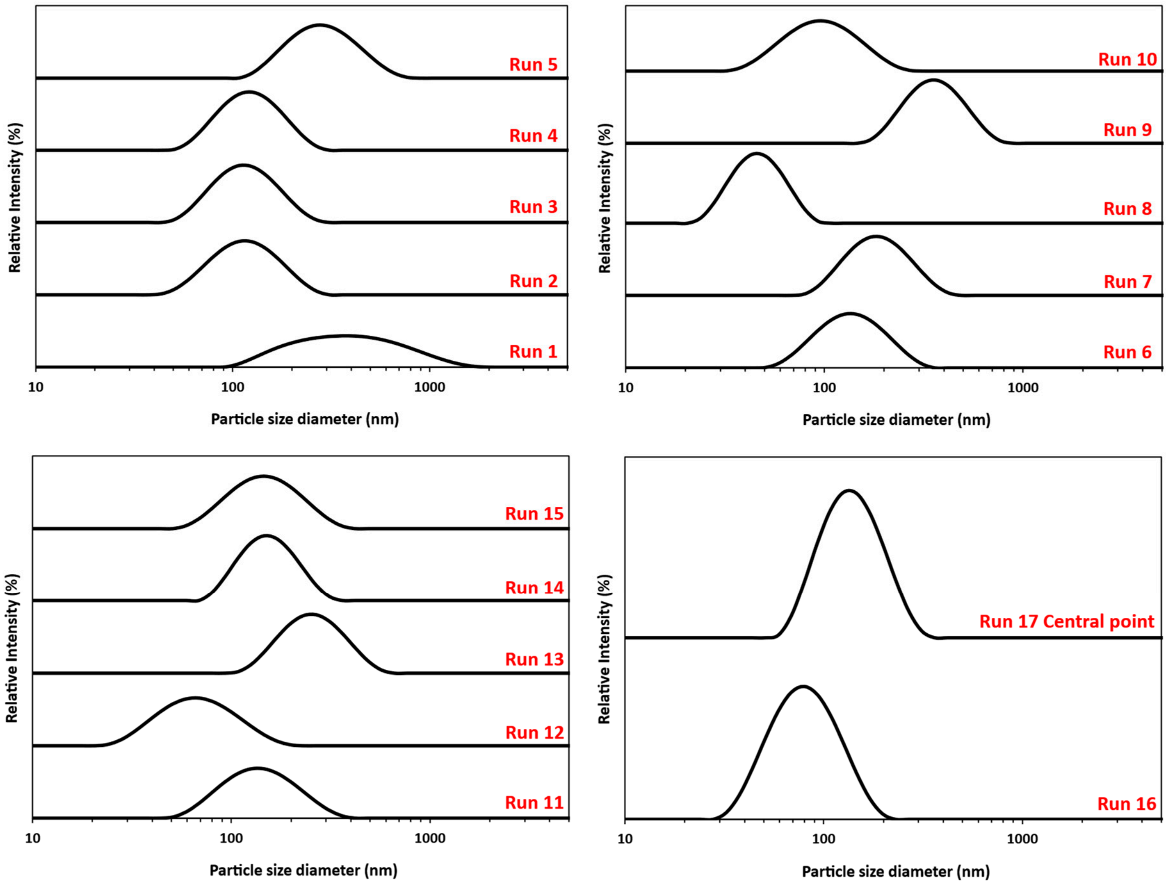

Figure S8: Particle size distribution in terms of relative scattered intensity from DLS according to the experimental design runs.

Coded Coefficients

Term	Effect	Coef	SE Coef	T-Value	P-Value	VIF
Constant		155,50	1,39	112,25	0,000	
x1	23,88	11,94	1,39	8,62	0,000	1,00
x2	-4,40	-2,20	1,39	-1,59	0,156	1,00
x3	-121,00	-60,50	1,39	-43,67	0,000	1,00
x4	-95,82	-47,91	1,39	-34,59	0,000	1,00
x5	-45,65	-22,83	1,39	-16,48	0,000	1,00
x1*x2	-7,83	-3,91	1,39	-2,82	0,026	1,00
x2*x3	-7,80	-3,90	1,39	-2,82	0,026	1,00
x2*x5	6,40	3,20	1,39	2,31	0,054	1,00
x3*x4	55,28	27,64	1,39	19,95	0,000	1,00
x3*x5	16,90	8,45	1,39	6,10	0,000	1,00
x4*x5	10,02	5,01	1,39	3,62	0,009	1,00
Ct Pt		-21,40	3,10	-6,91	0,000	1,00

Model Summary

S	R-sq	R-sq(adj)	R-sq(pred)
5,54098	99,82%	99,52%	97,96%

Analysis of Variance

Source	DF	Adj SS	Adj MS	F-Value	P-Value
Model	12	121870	10155,9	330,78	0,000
Linear	5	105987	21197,4	690,41	0,000
x1	1	2280	2280,1	74,26	0,000
x2	1	77	77,4	2,52	0,156
x3	1	58564	58564,0	1907,47	0,000
x4	1	36730	36729,7	1196,31	0,000
x5	1	8336	8335,7	271,50	0,000
2-Way Interactions	6	14418	2403,0	78,27	0,000
x1*x2	1	245	244,9	7,98	0,026
x2*x3	1	243	243,4	7,93	0,026
x2*x5	1	164	163,8	5,34	0,054
x3*x4	1	12221	12221,3	398,06	0,000
x3*x5	1	1142	1142,4	37,21	0,000
x4*x5	1	402	402,0	13,09	0,009
Curvature	1	1465	1465,5	47,73	0,000
Error	7	215	30,7		
Lack-of-Fit	4	148	37,0	1,66	0,354
Pure Error	3	67	22,3		
Total	19	122085			

Regression Equation in Uncoded Units

Particle size average (nm) = 1261,7 + 3,202 x1 + 0,823 x2 - 11,394 x3 - 40,46 x4 - 0,2601 x5 - 0,01597 x1*x2 - 0,00796 x2*x3 + 0,000154 x2*x5 + 0,3685 x3*x4 + 0,001988 x3*x5 + 0,001573 x4*x5 - 21,40 Ct Pt

Figure S9: Summary of the experimental design results with the coded coefficients, the model summary, the analysis of the variance and the regression equation concerning x_1 (initial lignin concentration, g/L), x_2 (solvent flow rate, ml/min), x_3 (antisolvent composition, water/EtOH, v/v), x_4 (antisolvent ratio, solvent/antisolvent, v/v), x_5 (antisolvent stirring speed, rpm) and their alias.

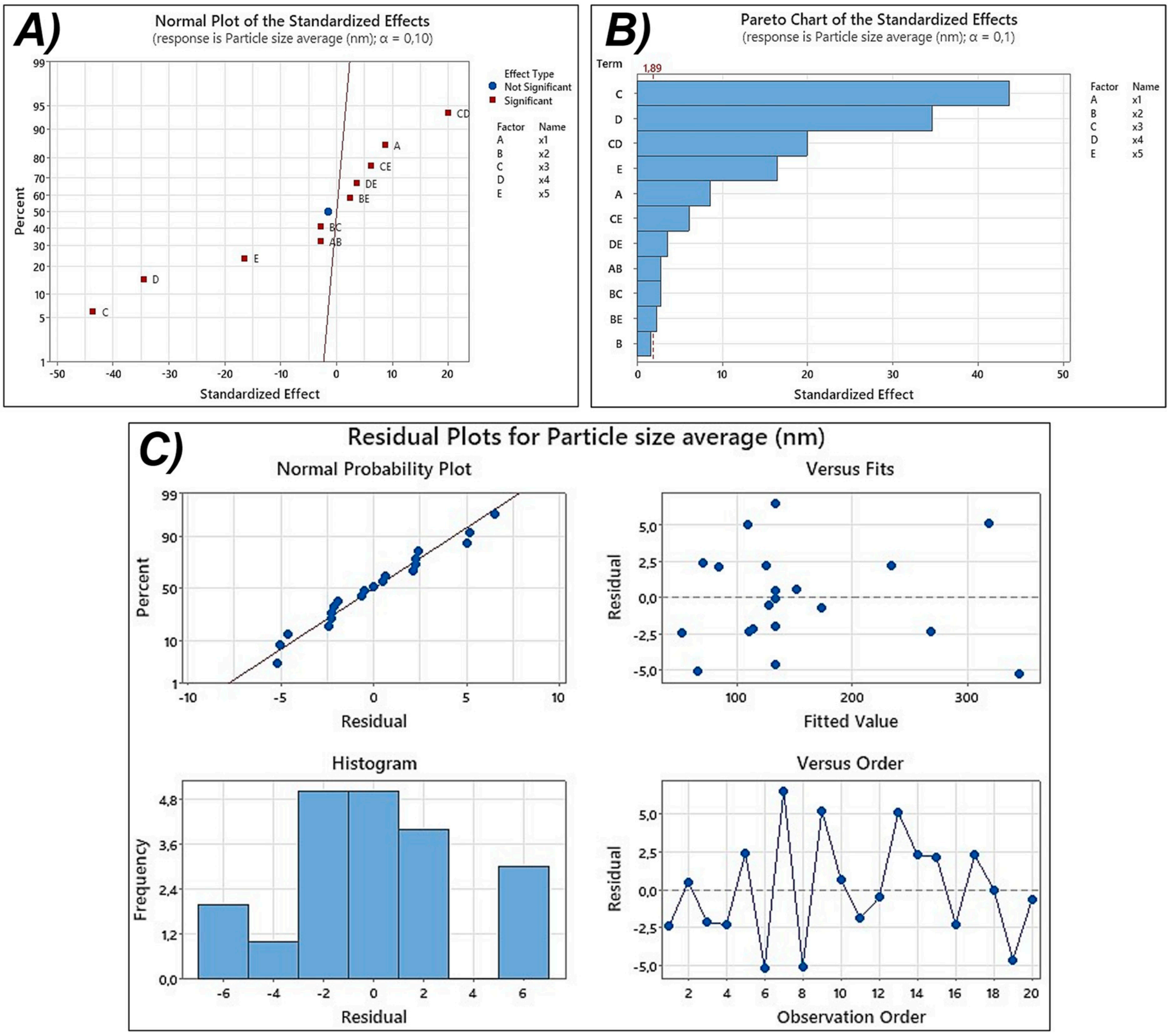

Figure S10: The experimental design results concerning x_1 (initial lignin concentration, g/L), x_2 (solvent flow rate, ml/min), x_3 (antisolvent composition, water/EtOH, v/v), x_4 (antisolvent ratio, solvent/antisolvent, v/v), x_5 (antisolvent stirring speed, rpm) and their alias. A) The normal plot of the standardised effects according to the response (particle size average, nm). All factors appear significant except for x_2 (B, in blue). B) The pareto chart of the standardized effects according to the response (particle size average, nm). All factors appear significant except for x_2 (B, value below the lower threshold). C) The residual plots for the response (particle size average, nm).

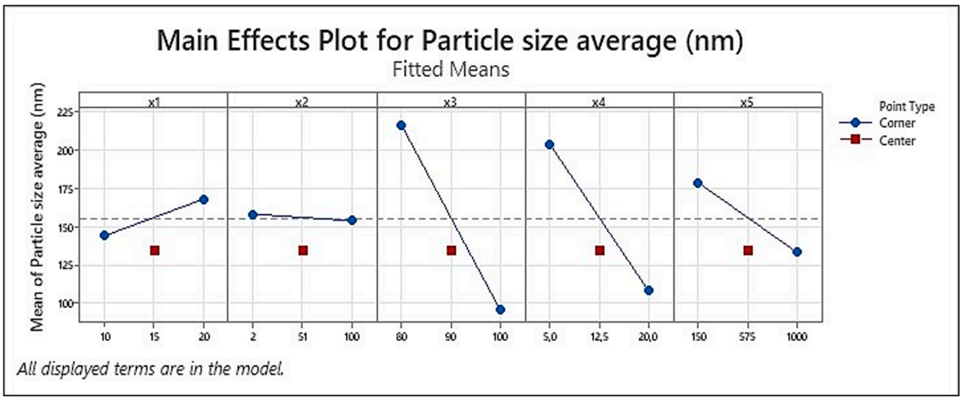

Figure S11: The main effects plot for the response (particle size average, nm), concerning x_1 (initial lignin concentration, g/L), x_2 (solvent flow rate, ml/min), x_3 (antisolvent composition, water/EtOH, v/v), x_4 (antisolvent ratio, solvent/antisolvent, v/v), and x_5 (antisolvent stirring speed, rpm). All factors appear to be significant.

References:

1. He, Q.; Ziegler-Devin, I.; Chrusciel, L.; Obame, S.N.; Hong, L.; Lu, X.; Brosse, N. Lignin-First Integrated Steam Explosion Process for Green Wood Adhesive Application. *ACS Sustainable Chem. Eng.* **2020**, *8*, 5380–5392, doi:10.1021/acssuschemeng.0c01065.
2. Girard, V.; Chapuis, H.; Brosse, N.; Canilho, N.; Marchal-Heussler, L.; Ziegler-Devin, I. Lignin Nanoparticles: Contribution of Biomass Types and Fractionation for an Eco-Friendly Production. *ACS Sustainable Chem. Eng.* **2024**, *12*, 7055–7068, doi:10.1021/acssuschemeng.4c00711.
3. Constant, S.; Wienk, H.L.J.; Frissen, A.E.; Peinder, P.D.; Boelens, R.; Van Es, D.S.; Grisel, R.J.H.; Weckhuysen, B.M.; Huijgen, W.J.J.; Gosselink, R.J.A.; et al. New Insights into the Structure and Composition of Technical Lignins: A Comparative Characterisation Study. *Green Chem.* **2016**, *18*, 2651–2665, doi:10.1039/C5GC03043A.
4. Steinmetz, V.; Villain-Gambier, M.; Klem, A.; Gambier, F.; Dumarcay, S.; Trebouet, D. Unveiling TMP Process Water Potential As an Industrial Sourcing of Valuable Lignin–Carbohydrate Complexes toward Zero-Waste Biorefineries. *ACS Sustainable Chem. Eng.* **2019**, *7*, 6390–6400, doi:10.1021/acssuschemeng.9b00181.
